# Treatments used for malaria in young Ethiopian children: a retrospective study

**DOI:** 10.1186/s12936-018-2605-x

**Published:** 2018-12-05

**Authors:** Abyot Endale Gurmu, Teresa Kisi, Habteweld Shibru, Bertrand Graz, Merlin Willcox

**Affiliations:** 1 Department of Pharmacognosy, School of Pharmacy, College of Medicine and Health Sciences, University of Gondar, Gondar, Ethiopia; 2 Department of Public Health, College of Health Sciences, Arsi University, Asella, Ethiopia; 3 Department of Internal Medicine, College of Medicine and Health Sciences, University of Gondar, Gondar, Ethiopia; 4 Antenna Foundation, Geneva, Switzerland; 5 Department of Primary Care and Population Sciences, Aldermoor Health Centre, University of Southampton, Aldermoor Close, Southampton, SO16 5ST UK

## Abstract

**Background:**

In Ethiopia, medicinal plants have been used to treat different diseases, including malaria, for many centuries. People living in rural areas are especially noted for their use of medicinal plants as a major component of their health care. This study aimed to study treatment-seeking and prioritize plants/plant recipes as anti-malarials, in Dembia district, one of the malarious districts in Northwest Ethiopia.

**Methods:**

Parents of children aged under 5 years who had had a recent episode of fever were interviewed retrospectively about their child’s treatment and self-reported outcome. Treatments and subsequent clinical outcomes were analysed using Fisher’s exact test to elicit whether there were statistically significant correlations between them.

**Results and discussion:**

Of 447 children with malaria-like symptoms, only 30% took the recommended first-line treatment (ACT) (all of whom were cured), and 47% took chloroquine (85% cured). Ninety-nine (22.2%) had used medicinal plants as their first-choice treatment. *Allium sativum* (Liliaceae), *Justicia schimperiana* (Acanthaceae), *Buddleja polystachya* (Scrophulariaceae*)* and *Phytolacca dodecandra* (Phytolaccaceae) were the most frequently used. *Justicia schimperiana* was the one associated with the best clinical outcomes (69% self-reported cure rate). However, the difference in clinical outcomes between the plants was not statistically significant.

**Conclusion:**

In this study, only 30% of children took the recommended first-line treatment. 22% of children with presumed malaria were first treated with herbal medicines. The most commonly used herbal medicine was garlic, but *J. schimperiana* was associated with the highest reported cure rate of the plants. Further research is warranted to investigate its anti-malarial properties.

## Background

Ethiopia has made tremendous progress in reducing incidence and mortality from malaria. There was a rapid scale-up of long-lasting insecticidal nets (LLINs) and artemisinin-based combination therapy (ACT) in 2007, associated with a 73% reduction in malaria admissions and a 62% reduction in malaria deaths in children aged under 5 years [1]. The Health Extension Programme increased coverage of primary health care in Ethiopia to 90% in 2010 [2]. Health extension workers are employed, each of whom is responsible for 500 households. Amongst other tasks, they can conduct rapid diagnostic tests for malaria and administer anti-malarial drugs. This contributed to Ethiopia’s rapid reduction in under 5 mortality, the fastest in East Africa, from 205 deaths per 1000 live births in 1990 to 64 in 2013 [3].

Because of increasing chloroquine resistance, in 2004 Ethiopia adopted artemether–lumefantrine (AL) as the first-line treatment for *Plasmodium falciparum* infections. Chloroquine remains the first-line treatment for *Plasmodium vivax*. Although national treatment guidelines recommend that this should be followed by a 2-week course of primaquine, in practice it is not routinely used because there are no widely available tests for G6PD deficiency. It is only used under the supervision of healthcare providers for patients with limited risk of malaria infection in the future, such as those who are not living in malaria endemic areas. A recent review showed that in Ethiopia, 98.1% of patients with *P. falciparum* were successfully treated with AL and 94.7% of patients with *P. vivax* were successfully treated with CQ [4]. However, AL is only effective in 75.1% of patients with *P. vivax* [4]. Although prevalence of *P. falciparum* has reduced rapidly since the introduction of AL, prevalence of *P. vivax* has risen slightly [5].

Therefore, malaria remains a leading public health problem in Ethiopia. It is estimated that about 75% of the total area of the country and 65% of the population is at risk of infection [6]. In 2016, an estimated 2.6 million malaria cases and 5000 deaths occurred in the country, which was an increase since 2014 [7]. Two-thirds of cases were due to *P. falciparum*, but Ethiopia is home to the second highest number of cases and mortality due to *P. vivax* in the world (after India). There is still room for progress: use of insecticide-treated bed nets in children under 5 ranges from 45 to 69% [8, 9]. The biggest gap between practice and policy is that only 41% of children under 5 with fever were taken to a health facility or provider, and only 34% sought care promptly [9]. Furthermore, AL was less than 20% of the anti-malarials received by under-5 s [7].

One possible explanation for this is the use of herbal medicines. Traditional medicinal plants are an integral part of the variety of cultures in Ethiopia [10]; up to 80% of the population uses traditional medicine due to the cultural acceptability of healers and local pharmacopeias, the relatively low cost of traditional medicine and difficult access to modern health facilities [11]. People living in rural areas are particularly noted for their use of medicinal plants as a major component of their health care [12–15]. The hypothesis of the current study was that many people who do not use formal care are using herbal medicines instead. Plants which are traditionally used for the treatment of malaria are a potential source of active lead compounds with new mechanisms of action.

The classical way of identifying medicinal plants for further research is through ethnobotanical studies. Yet conventional ethnobotanical studies rarely involve clinicians. They could and should provide much more clinical information if the ultimate goal is to know which one, among numerous treatments for a given ailment, has the best effects. Although identification of the plants is usually of a good standard, definition of the diseases which they treat is not. There is rarely sufficient questioning about the observed patient status and progress, perceived efficacy and limitations of the remedies, and whether these are indeed the ‘treatment of choice’. Many plants are ‘supposed’ to be good for one disease or another, but are not actually the preferred treatment used in everyday life. The ‘Retrospective Treatment Outcome Study’ (RTO) was designed circumvent these problems [16]. This adds two essential elements to the ethnobotanical method: clinical information and statistical analysis. Clinical information is collected retrospectively on the presentation and progress of a defined disease episode. This approach has proved to work well in Mali [17], and has then been used in other places.

### Aims and objectives

This study aimed to measure the frequency of use of different treatments, and associated outcomes, in children under 5 years of age with a recent episode of fever (identified by the parents as uncomplicated malaria) in a rural district of Ethiopia.

## Methods

### Study site

**Fig. 1:**
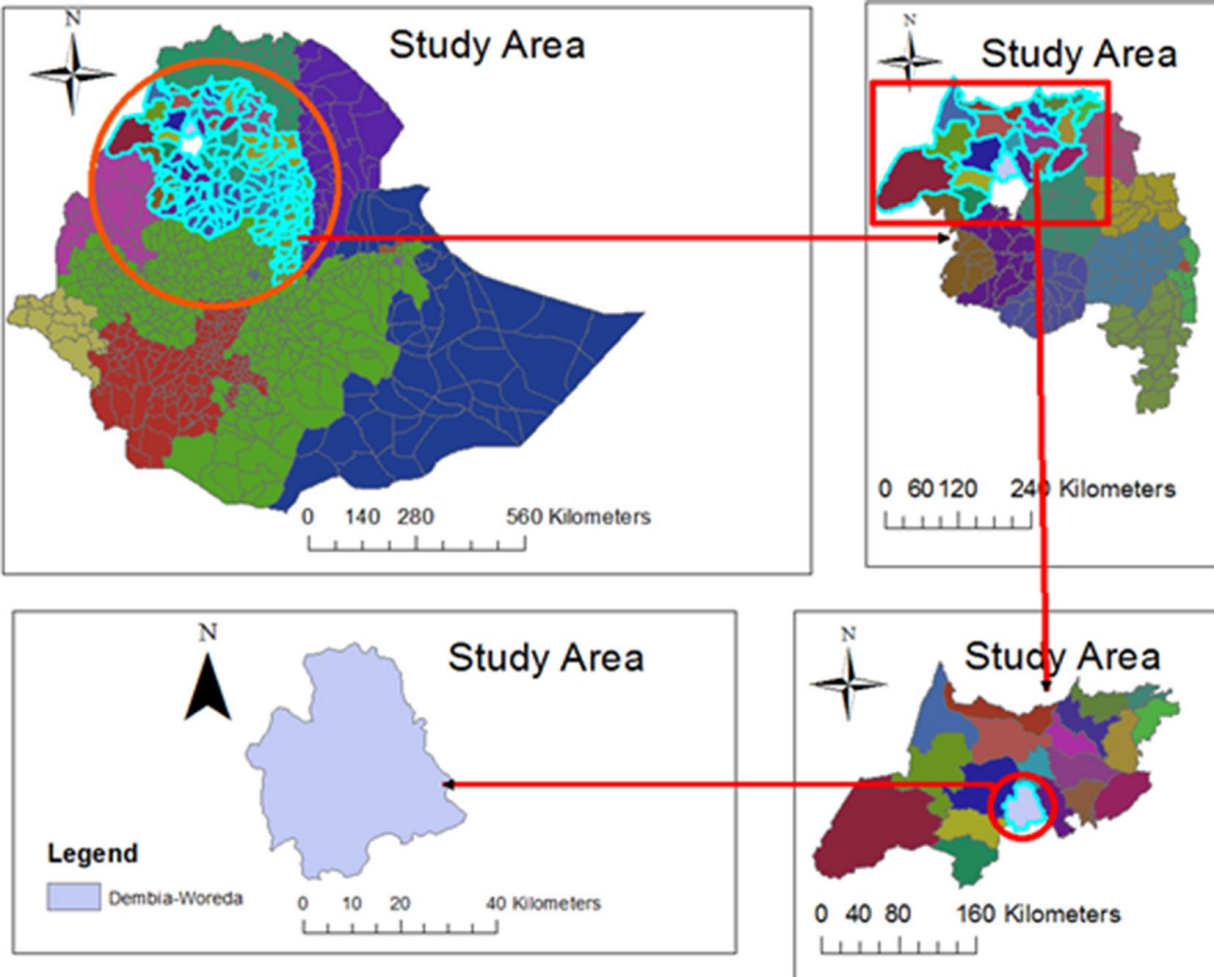
Map of the study area

Dembia is a rural district in Northwest Ethiopia, covering a total area of 127,000 km^2^ (Fig. 1). The altitude of the district ranges from 1750 to 2100 m above sea level. It lies next to the largest lake in Ethiopia (Lake Tana) which contributes to the high and rising level of malaria in the area [18]. Following the rainy season, the incidence of malaria peaks from the end of September to the middle of December. There were 12,221 malaria cases in the district in the year 2012, and this rose to 22,166 in 2016 [18]. Within the district there are 5 urban *kebeles* and 40 rural *kebeles* (a *kebele* is the smallest administration unit, which is equivalent to a village). According to demographic data of the district, Aberjeha, Chenker and Tezeba were the most malarious kebeles with populations of 10,490, 6520 and 6213, respectively, and with incidence rates of 7.7%, 7.0% and 6.6%, respectively (Dembia District Health Office Demographic Data, 2012). These were selected for the study. The proportion of under five children was estimated to be 14% (3251) of the total population.

### Sampling

The sample size for the study was determined using the formula for a single population proportion. It was estimated that 50% of the population would be using plants, and required a 95% level of confidence. Therefore, sample size was determined as follows:

$$ {\text{n }} = \, \frac{{\left( {{\text{Z}}_{\alpha / 2} } \right)^{ 2} {\text{P }}\left( { 1- {\text{ P}}} \right)}}{{{\text{d}}^{2} }} \, = \, \frac{{\left( { 1. 9 6} \right)^{ 2} 0. 5\left( {0. 5} \right)}}{{(0.05)^{2} }} \, = { 385} $$

In order to allow for an estimated 15–17% non-response, the final sample size was increased to 451. The selection of respondents was performed in order to ensure that they were representative of the whole population, with a corresponding proportion of houses to be randomly visited until the desired sample size was reached.

### Data collection process

During the peak malaria season, from November to December 2013, the parents of children aged under 5 years who had had symptoms of uncomplicated malaria (mainly fever) within the previous 2 months were interviewed. Data was collected using a structured and pre-tested questionnaire which was developed by Graz et al. [16] and subsequently modified, in order to elicit relevant symptoms, treatments and self-reported outcomes. Specimens of the reported anti-malarial plants were collected and identified by Mr. Abiyu Enyew (Botanist), Department of Biology, College of Natural Sciences, University of Gondar, to correspond the local name to the scientific name and deposited at herbarium unit, Department of Biology.

### Data analysis

Data were coded, checked for completeness and consistency, entered using EPI-INFO™7 statistical software and then exported to SPSS version 20 for further analysis. Descriptive statistics of the collected data (list of plants/recipes used, frequency of children used, mode of preparation and treatment outcome, form of the treatment) was done. Statistical correlation with reported clinical recovery was also computed using Fisher’s exact test.

### Permissions and ethical clearance

The study was carried out after getting permission from the ethical review board of the University of Gondar (R/C/S/V/P/05/239/2013). Then a letter of permission was obtained from the district health office and local administrator, and the individual household heads were invited to give written informed consent before the interview. Confidentiality was respected by keeping the privacy of the respondents while filling the questionnaire. Seriously ill children were advised to visit a health facility. Any personally identifiable information (such as names, addresses) was not entered into the database.

## Results

### Socio-demographic characteristics of respondents

Among 451 patients who were approached for the study, 4 either did not complete the interview or were not willing to be included in the study. Finally, 447 were included, resulting in a response rate of 99.1%. 52.1% were girls and half of them (50.3%) were aged between 1 and 3 years. The mean age was found to be 34 months, with a range between 7 months and 5 years.

### Treatment providers and treatment choices

Over three-quarters of respondents (75.6%) had sought treatment from a nurse, health extension worker, doctor or pharmacist, mostly (50.1%) from a nurse (Table 1). For those who took herbal medicines, more were provided by a family member (13.5%) than by a traditional healer (8.5%).

**Table 1:** Treatment providers for children under 5 years of age with fever in Dembia district, Northwest Ethiopia

Treatment providers	Frequency (percentage)
Nurses	224 (50.1%)
Health extension workers	56 (12.5%)
Pharmacist/druggist	49 (11%)
Doctors	2 (0.4%)
Drug vender	16 (3.6%)
Traditional healers	38 (8.5%)
Family	60 (13.4%)
Not mentioned	2 (0.4%

**Fig. 2:**
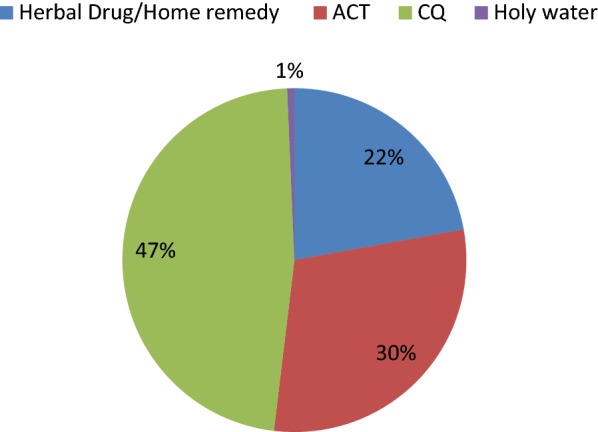
First line treatment for malaria and malarial like symptoms in under five children

As shown in Fig. 2, from the total of 447 children with malaria, the commonest treatment was chloroquine (47%) followed by ACT (30%), usually artemether + lumefantrine. Ninety-nine (22.2%) were found to use medicinal plants alone for treatment of the illness as a first choice while 12 and 3 were found to use medicinal plants as a second and third alternative, respectively. Holy water was also reported to be used by three respondents as the first choice. Twelve plant species were mentioned and identified as treatments for malaria. *Allium sativum* (Liliaceae) was the most frequently reported plant (Table 2).

**Table 2:** Traditional recipes and patient-reported clinical outcomes in children under 5 years of age with fever in Dembia district, Northwest Ethiopia

Plant species used	Plant part	Preparation	No of cases reporting use	No of cases reporting clinical recovery (%)		No of treatment failures
				Cured	Improved	
*Allium sativum* (Liliaceae)	Bulb	Decoction	35	18 (53%)	17 (49%)	0
*Justicia schimperina* (Hochst) Dandy. (Acanthaceae).	Leaf	Maceration	16	11 (69%)	4 (25%)	1 (6%)
*Buddleja polystachya* (Scrophulariaceae*)*	Leaf	Maceration/Infusion	16	9 (56%)	6 (38%)	1 (6%)
*Phytolacca dodecandra* (Phytolaccaceae)	Root	Maceration/decoction	16	8 (50%)	8 (50%)	0
*Verbena officinalis* (Verbenaceae)	Leaf/aerial part	Maceration	5	3 (60%)	2 (40%)	0
*Ocimum lamiifolium* (Lamiaceae)	Leaf	Infusion	5	2 (40%)	3 (60%)	0
*Allium sativum* (Liliaceae) +* Brassica nigra* (Cruciferaceae)	Bulb/seed	Maceration	3	2	1	0
*Brassica nigra* (Cruciferaceae) + Honey	Seed	Decoction	2	1	1	0
*Zehneria scabra* (Cucurbitaceae)	Arial part	Inhalations	2	1	1	0
*Zingiber officinale (*Zingiberaceae)	Rhizome	Infusion	2	1	1	0
*Achyranthes aspera* L. (Amaranthaceae)	Leaf	Infusion	1	1	0	0
*Clerodendrum myricoides* (Lamiaceae)	Leaf	Maceration	1	1	0	0
*Allium sativum* (Liliaceae) + *Verbena officinalis* (Verbenaceae)	Bulb/aerial part	Decoction	1	1	0	0
Honey + *Allium sativum* (Liliaceae)	Bulb	Infusion	1	0	1	0
*Ocimum lamiifolium* (Lamiaceae) + *Zehneriascabra* (Cucurbitaceae)	Leaf/aerial part	Infusion	1	0	1	0
Honey + *Brassica nigra* (Cruciferaceae)		Infusion	2	1	1	0
Total			109	60 (55.0%)	47 (43.1%)	2 (1.8%)

### Treatments used and associated outcomes

According to their parents, all children who were treated with ACT were cured while 84.6% and 15.4% of children treated with CQ were cured and improved, respectively (p < 0.001). Of the herbal treatments, garlic was the most commonly used, but *Justicia schimperiana* was associated with the highest proportion of patients who said they were “cured” (69%). Although this seemed to be higher than the reported cure rate for all other medicinal plants (53%), the difference was not statistically significant (Fisher’s exact test statistic = 0.284).

Most patients used leaves (41.9%) followed by roots (17.1%). The oral route was the most frequent form of administration (92.8%) whereas decoctions were the most common preparation. Respondents were also asked about the unit of measurement for medicinal preparations and the majority of them used teaspoons and coffee cups for liquid preparations such as decoctions. Water was the most widely used solvent and honey was commonly used as sweetening agent to mask unpleasant tastes.

## Discussion

### Summary of findings and comparison with the literature

It was found that much higher rates of treatment-seeking from formal health workers than reported in other sources. In spite of this, only a minority of children with malaria were treated with ACT, confirming results of other studies [7]. Chloroquine was the commonest treatment, used in almost half of all cases. This is more frequent than would be expected, given that *P. vivax* is estimated to cause less than one-third of malaria cases in Ethiopia [7]. This may in part be because CQ can be obtained from drug shops at a relatively cheap price ACT are only available from official health centres.

Although there are a few reports of CQ resistant *P. vivax* in Ethiopia [19–22], CQ is still recommended for the treatment of *P. vivax* malaria in Ethiopia [23] and is still effective in 94.7% of cases [4]. The lower reported “cure” rate in this study (reported by parents) could imply that chloroquine was being used for cases of falciparum malaria (some of which are resistant to chloroquine), and that some of the children may have had a disease other than malaria.

The use of herbal medicine was lower than expected. This may partly be due to the expansion of the Health Extension Programme (HEP) at the household level which increased treatment-seeking for malaria in some areas [24] although in this study, only 12.5% of respondents received treatment from a Health extension worker.

None of the plants were associated with a very high reported “cure” rate, unlike studies in some other African countries [17]. However almost all parents reported that their children had “improved”. Previous ethnobotanical studies have shown that several plants reported here are used elsewhere for the management of malaria and malaria-like symptoms (Table 3).

**Table 3:** Previous ethnobotanical reports of anti-malarial use of the most frequently cited plants

Plant	Part used	Preparation	Country	References
*Allium sativum*	Bulb	Maceration in oil, Swallowing (eating the bulb)	India, Nigeria, Ethiopia	[12, 14, 29–36]
*Brassica nigra*	Seed	Maceration	India, Ethiopia	[12, 37, 38]
*Buddleja polystachya*	Leaf	Juice	Ethiopia	[15]
*Justicia schimperiana*	Leaf	Maceration	Ethiopia	[12, 34, 39]
*Phytolacca dodecandra*	Leaf, root	Maceration	Ethiopia	[31, 34]
*Zingiber officinale*	Rhizome	Maceration, decoction, mixture	Nigeria, India, Sri Lanka, Zambia, Nicaragua	[30, 32, 40–43]
*Ocimum lamiifolium*	Leaf	Concoction	Ethiopia	[35]
*Clerodendrum myricoides*	Leaf, root	Maceration	Ethiopia	[34]

The anti-malarial activity of several of these plant extracts has been assessed in rodent models in vivo (Table 4). Ajoene, a compound isolated from *Allium sativum,* was found to prevent the development of parasitaemia in mice infected with *Plasmodium berghei* and substantially improved the anti-malarial activity of chloroquine [25]. However the most promising plant was *J. schimperiana.* Although not the most commonly used, it was associated with the highest reported cure rates in this study. Its root extract has been tested against *P. falciparum* in vitro and was not very active (IC50 = 71 mcg/ml for the methanolic extract) [26]. However, aqueous leaf extracts (which more closely resemble the traditional preparation) suppressed growth of *P. berghei* in mice in the 4-day suppressive test by 41%, and the methanol leaf extract suppressed parasitaemia by 65% [27]. Although *Clerodendrum myricoides* and *Zehneria scabra* also had good anti-malarial activity in mouse models, they were not widely used, possibly because they are widely regarded as poisonous [28].

**Table 4:** Previous studies on the in vivo anti-malarial activity of the most frequently cited plant extracts, given orally to mice (infected with *Plasmodium berghei*)

Plant name	Plant part	Type of extract	Dose (mg/kg)	% Chemosuppression	Reference
*Justicia schimperiana*	Leaf	Hydro-alcoholic	200, 400, 600	8, 65 and 85, respectively	[44]
*Justicia schimperiana*	Leaf	Chloroform	200, 400, 600	16, 26 and 28, respectively	[27]
		Methanol	200, 400, 600	37, 50 and 65, respectively	
		aqueous	200, 400, 600	18, 32 and 40, respectively	
*Allium sativum*	Isolated compounds	Ajoene	50	About 67%	[25]
		Allicin	9	94	[45]
*Brassica nigra*	Seed	Hydroalcoholic	100, 200,400	22, 50 and 53, respectively	[46]
*Phytolacca dodecandra*	Leaf	Hydroalcoholic	100, 200, 400	18, 51 and 55, respectively	[47]
*Zehneria scabra*	Leaf	Hydroalcoholic	100, 200, 400	62, 73 and 76, respectively	[48]
		Chloroform	25,50,100	53, 74 and 61, respectively	
		Ethyl acetate	25,50,100	72,62 and 73, respectively	
*Clerodendrum myricoides*	Leaf	Methanol	600	82.5	[49]

### Strengths and limitations of the study

This was a community-based study conducted in one of the most malarious areas of Ethiopia. There was a very high response rate so the results are likely representative of the population in this area, and it could be confident in the prevalence of use of the different treatments. Voucher specimens of plants were collected and identified by a botanist.

The first limitation of this study is that patients were not asked whether they had been tested before receiving the treatment. With hindsight, it would have been useful to know the proportion of patients tested, and the proportion with *P. falciparum* or *P. vivax*. As the survey was retrospective, it is likely that some patients were not tested, and some may not actually have had malaria. Secondly, parents may not have been aware of the qualifications of the person they saw and may have said she was a ‘nurse’ when in fact she may have not been qualified. Thirdly, the data on outcomes is based on self-reporting by parents of the children. It seems that the question which discriminated best between treatments was whether a patient was ‘cured’, since almost all patients claimed to have at least “improved”. Therefore, the % of patients ‘cured’ on each treatment was compared. This approach seems to be valid, because 100% of patients claim to have been ‘cured’ after taking an ACT (to which there is no documented resistance in Ethiopia) compared to 84.6% who took chloroquine, against which there is some resistance.

Lastly, because herbal medicine was less frequently used than predicted, the sample size was too small to be able to find statistically significant differences in outcomes between patients who had taken different herbal remedies. Sample size calculation was based on an estimation of 50% of patients having taken herbal medicines. If that had been the case, this sample would have included twice as many patients who had taken herbals, which would have increased the statistical power to find differences in outcomes between subgroups—for example those who had taken *J. schimperiana* versus those who had taken *Allium sativum*.

### Implications for policy, practice and research

Further investigations are needed to understand why the majority of children with malaria in Ethiopia receive CQ rather than AL, although the majority of malaria cases are reported to be caused by *P. falciparum* rather than *P. vivax*. Since most of the anti-malarial medicines were provided by health workers, a qualitative study of health workers would help to understand this. Factors to explore would include availability of AL and use of diagnostic tests or microscopy to distinguish between malaria species.

*Justicia schimperiana* may have the potential to be a candidate for the development of efficacious and safe anti-malarial phytomedicines and/or compounds, using a ‘reverse pharmacology’ model [17]. It would be useful to further investigate the way in which it is prepared and used, and to isolate the active phytochemical(s).

## Conclusion

In the most malarious villages of Dembia district, Ethiopia, only 30% of children with presumed malaria took the recommended first-line treatment (artemether–lumefantrine), while 47% took chloroquine and 22% were treated with herbal medicines as the first-line treatment. The most commonly used herbal medicine was garlic, but *J. schimperiana* was associated with the highest proportion of patients who said they were ‘cured’ (69%). Further research is warranted to understand reasons for the low use of AL, and to investigate the anti-malarial properties of *J. schimperiana*.

## Abbreviations

RTO: Retrospective Treatment Outcomes
SPSS: Statistical Packages for Social Sciences
